# Enhanced susceptibility of cancer cells to oncolytic rhabdo-virotherapy by expression of Nodamura virus protein B2 as a suppressor of RNA interference

**DOI:** 10.1186/s40425-018-0366-2

**Published:** 2018-06-19

**Authors:** Donald Bastin, Amelia S. Aitken, Adrian Pelin, Larissa A. Pikor, Mathieu J. F. Crupi, Michael S. Huh, Marie-Claude Bourgeois-Daigneault, John C. Bell, Carolina S. Ilkow

**Affiliations:** 1 Centre for Innovative Cancer Research, Ottawa Hospital Research Institute, Ottawa, K1H 8L6 Canada; 2 Department of Biochemistry, Microbiology and Immunology, University of Ottawa, Ottawa, K1H 8M5 Canada

**Keywords:** Oncolytic virus, RNA interference, Vesicular stomatitis virus, B2

## Abstract

Antiviral responses are barriers that must be overcome for efficacy of oncolytic virotherapy. In mammalian cells, antiviral responses involve the interferon pathway, a protein-signaling cascade that alerts the immune system and limits virus propagation. Tumour-specific defects in interferon signaling enhance viral infection and responses to oncolytic virotherapy, but many human cancers are still refractory to oncolytic viruses. Given that invertebrates, fungi and plants rely on RNA interference pathways for antiviral protection, we investigated the potential involvement of this alternative antiviral mechanism in cancer cells. Here, we detected viral genome-derived small RNAs, indicative of RNAi-mediated antiviral responses, in human cancer cells. As viruses may encode suppressors of the RNA interference pathways, we engineered an oncolytic vesicular stomatitis virus variant to encode the Nodamura virus protein B2, a known inhibitor of RNAi-mediated immune responses. B2-expressing oncolytic virus showed enhanced viral replication and cytotoxicity, impaired viral genome cleavage and altered microRNA processing in cancer cells. Our data establish the improved therapeutic potential of our novel virus which targets the RNAi-mediated antiviral defense of cancer cells.

**Electronic supplementary material:**

The online version of this article (10.1186/s40425-018-0366-2) contains supplementary material, which is available to authorized users.

## Background

Oncolytic viruses (OVs) possess an intrinsic or engineered ability to selectively target, replicate in and kill cancer cells [1]. These promising cancer therapeutics exploit cellular defects that promote tumour growth [2], destroy tumour-associated vasculature [3], induce anti-tumour immunity [1] and synergize with other treatments [4]. OVs must overcome barriers triggered by viral infection, including those mounted by cancer cells and components of the tumour microenvironment [5]. The type I interferon (IFN) pathway is a well-characterized mammalian signaling cascade triggered upon viral attack to protect the surrounding cells and alert the immune system to contain infection [6]. The production of type I IFNs promotes an antiviral and anti-proliferative state in addition to inducing innate and adaptive immunity [2]. This antiviral response represents a major barrier to virus replication and spread in healthy tissues and is necessary to the safety of OV therapy [7]. Interestingly, the genetic alterations promoting tumourigenesis are associated with enhanced cancer cell susceptibility to viral infection [2]. Many pathways activated in response to infection that inhibit cell growth, activate apoptosis, and alert the immune system, are incompatible with malignant growth and are often defective in cancer cells [2]. Since these defects are common in cancer cells, they facilitate the targeted killing of cancer cells by certain OVs. Despite having IFN pathway defects, many cancers are still quite resistant to OV therapy [7]. For example, the vesicular stomatitis virus (VSV) is an OV platform with promising potential for clinical translation [8]. A VSV variant with an improved therapeutic index (VSV∆51) is impaired in its capacity to block the IFN response and infect normal tissues [7]. The degree of susceptibility to VSV∆51 killing varies among human cancers [7], due to the IFN status of the cancer cells and the potential involvement of other antiviral mechanisms within resistant tumours.

An alternative antiviral strategy relies on RNA interference (RNAi) [9], in order to combat infection in plants, fungi and invertebrates. This system is similar to the microRNA (miRNA) processing pathway used for post-transcriptional regulation in most eukaryotes. Viral double-stranded RNA generated during replication and transcription is bound and cleaved by the host cytoplasmic enzyme Dicer to form 22–23 nucleotides long RNA fragments [10]. These short RNA fragments are loaded into the RNA-induced silencing complex (RISC) where a single strand is selected to target homologous viral RNA and therefore prevent viral genome replication and translation [10]. To counteract this RNAi-mediated antiviral response, many plant and insect viruses have evolved viral suppressors of RNAi (VSRs) [11]. One such virus is the Nodamura virus, which primarily infects insects but is also highly virulent to certain mammals like suckling mice and hamsters [11–13]. Nodamura virus encodes a VSR known as B2, which binds double-stranded RNA and inhibits processing by Dicer which prevents the production of antiviral siRNAs [14–16].

RNAi- and protein-mediated immunity were long thought to be non-overlapping mechanisms, with insects and invertebrates using one strategy and mammals using the other. Interestingly, recent discoveries suggest that these mechanisms might not be mutually exclusive. In fact, antiviral RNAi has been shown to function in mammalian embryonic or undifferentiated cells [17]. Considering the discovery of mammalian antiviral RNAi in embryonic stem cells and the genetic similarities between cancer cells and embryonic stem cells [18–22]; we hypothesized a role for antiviral RNAi in cancer cells. To investigate antiviral RNAi and its effects on OV therapy, we engineered a recombinant VSV∆51 to express the Nodamura virus B2 protein. Herein, we characterize this novel OV and demonstrate the interplay between the B2-expressing virus and RNA processing pathways in cancer. Our results show enhanced cancer-specific killing by our virus as well as improved viral replication in vivo. Together, our data strongly suggest the involvement of the RNAi pathway in the antiviral defense of cancer cells.

## Results

### Evidence of a functional antiviral RNAi mechanism detected in VSV∆51 infected cancer cells

**Fig. 1:**
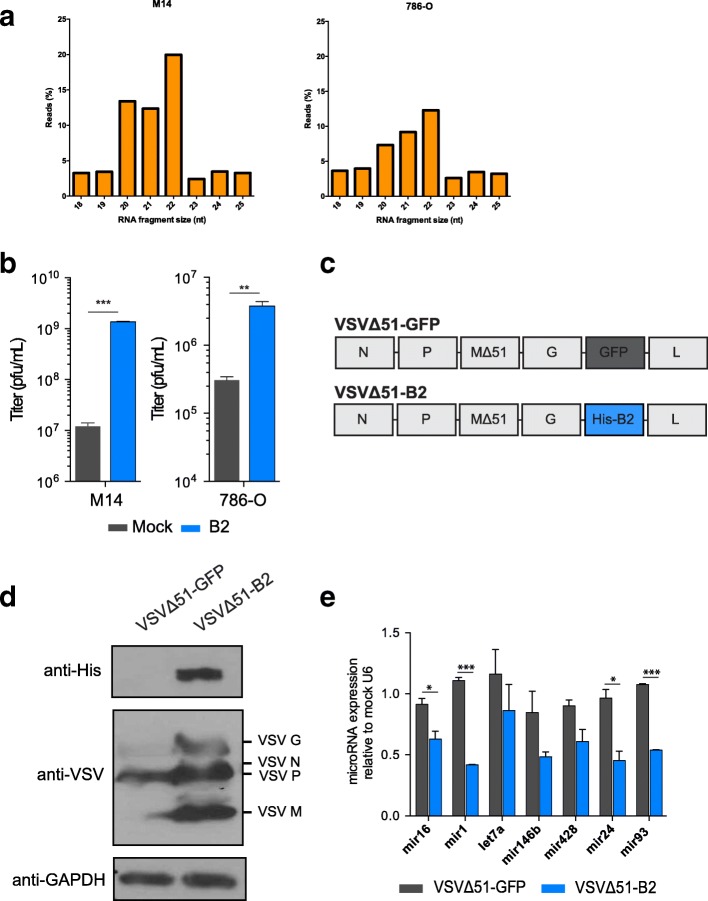
B2 enhances VSV∆51 replication and alters miRNA levels in cancer cell lines. **a** M14 or 786-O cells were infected with VSVΔ51 virus and small-RNA deep sequencing was performed. Virus-derived small RNAs have a length bias towards 22-mers. The enrichment for 22-mers is indicated for positive strand vsRNAs. **b** Virus concentrations of supernatants collected from M14 or 786-O cells expressing fluorescently-tagged B2 or empty vector and infected with VSVΔ51 at an MOI of 0.1 for 24 h. NS: *P* > 0.1; *P < 0.1, **P < 0.01, ***P < 0.001, using Student’s *t*-test. Only significantly different pairs are indicated. **c** Schematic representation of the VSV∆51-B2 and VSV∆51-GFP viral backbones. **d** Western blot analysis of Vero cells infected at an MOI of 1 with VSVΔ51 or VSVΔ51-B2 for 24 h. The membranes were probed for VSV proteins, His-tagged B2 and GAPDH. **e** MiRNA levels from 786-O cells infected with VSVΔ51-GFP or VSVΔ51-B2 for 18 h as determined by qPCR. The results were normalized to mock uninfected levels as explained in the material and methods section. NS: *P* > 0.1, *P < 0.1, **P < 0.01, ***P < 0.001, using Student’s *t*-test. Only significantly different pairs are indicated on the figure

To investigate the potential involvement of antiviral RNAi mechanisms in cancer cells, we infected human cancer cell lines with a VSV variant impaired in its ability to block IFN response (VSVΔ51) and performed small-RNA deep sequencing. We showed that virus-derived small RNAs (vsRNAs) have a length bias towards 22-mers in several cell lines (Fig. 1a and Additional file 1: Figure S1A), consistent with the size of Dicer cleavage products. Importantly, this enrichment for 22-mers is present in positive strand vsRNAs, suggesting the occurrence of double stranded RNA cleavage. This is also characteristic of Dicer products, and likely acts during the synthesis of positive strands in VSV genome replication.

### B2 protein enhances VSV∆51 replication in virus-resistant cancer cells

We reasoned that if antiviral RNAi is triggered in mammalian cancer cells upon VSV∆51 virus infection, expression of an RNAi viral suppressor should significantly enhance virus growth and cytotoxicity. To this end, we investigated the effects of the VSR protein B2 on VSV∆51 replication in cancer cells, and characterized two human cancer cell lines (melanoma M14 cells and renal carcinoma 786-O cells) transfected with fluorescently-tagged B2 or empty vector (mock control). M14 and 786-O cell lines were selected as models for further characterization as they are both resistant to VSV infection and have functional type I IFN pathways. Upon drug-selection and sorting of positive cells, we confirmed ectopic expression of fluorescently-tagged constructs, as shown in fluorescence microscopy images (Additional file 1: Figure S1B). Viral outputs from B2-expressing M14 and 786-O cells infected with VSV∆51 were significantly higher relative to mock controls (Fig. 1b), suggesting that B2 may enhance viral production.

### VSV∆51-mediated expression of B2 enhances cytotoxicity in cancer cell lines

As ectopic expression of the B2 protein enhanced VSV∆51 titers in both M14 and 786-O cell lines, we engineered a VSV∆51 virus variant encoding His-tagged B2 to assess effects of virus-mediated B2 expression. B2, or GFP as a control, was cloned between the G and L genes of the VSV∆51 backbone (Fig. 1c), using a strategy previously shown to support expression of transgenes without impairing virus replication [7, 23]. We infected Vero cells with VSVΔ51-B2 and confirmed transgene expression by immunoblotting for His-tagged B2. As predicted, expression of B2 enhanced expression of VSV viral proteins (Fig. 1d).

While the mechanism of action of VSVΔ51-encoded B2 on mammalian cancer cells remains to be elucidated, previous studies have shown that B2 blocks processing of small RNAs by Dicer [24, 25]. Given that B2 enhanced VSV∆51 production by mammalian cancer cells, we investigated whether miRNA levels were affected by VSV∆51-B2 using quantitative PCR (qPCR) for various miRNAs from infected 786-O cells. For the majority of miRNAs tested, including miR-1, miR-16, miR-24, and miR-93, the miRNA expression levels measured in VSV∆51-B2 infected samples were significantly lower compared to VSV∆51-GFP samples (Fig. 1e), suggesting inhibition of small RNA processing by B2.

**Fig. 2:**
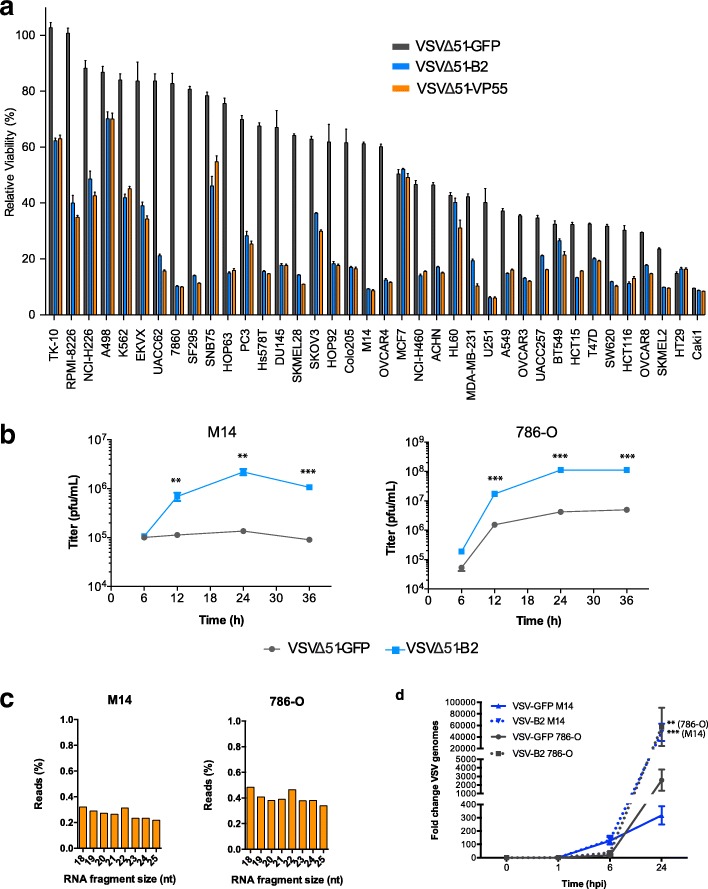
VSV∆51-B2 alters cytotoxicity and viral genome cleavage. **a** Relative metabolic activity of 38 human cancer cell lines infected with VSVΔ51-GFP or VSVΔ51-B2 or additionally VSVΔ51-VP55 for 48 h at an MOI of 1. The results are expressed as a percentage of the signal obtained compared to mock treatment. **b** Time-course of viral titers from 786-O and M14 cell lines infected with VSVΔ51-GFP or VSVΔ51-B2 at an MOI of 3. NS: *P* > 0.1, *P < 0.1, **P < 0.01, ***P < 0.001, using Student’s *t*-test. Only significantly different pairs are indicated on the fig. **c** We performed small-RNA deep-sequencing using M14 or 786-O cells infected with VSVΔ51-B2 at an MOI of 0.1 for 18 h. B2 expression in VSVΔ51 virus abrogates genomic cleavage as 22-mer vsRNAs are no longer prominent. VSVΔ51-B2 derived vsRNAs display a bias towards positive strand reads in M14 and 786-O cells. **d** The indicated human cancer cell lines were infected with VSVΔ51-GFP or VSVΔ51-B2 (MOI = 0.1). At the indicated time points, the expression level of virus genomes for each sample was quantified and normalized to *GAPDH*. Levels of VSV genomes are expressed relative to the level observed in the VSVΔ51-GFP 1 h-post-infection samples, which were arbitrarily set to 1. Error bars indicate ±SD among triplicates. NS: *P* > 0.1, *P < 0.1, **P < 0.01, ***P < 0.001, using Student’s *t*-test

To determine if VSV∆51-B2 could kill cancer cells more efficiently than VSV∆51-GFP, we screened a panel of 38 different human cancer cell lines. The cells were infected at a multiplicity of infection (MOI) of 1 and cell viability was assessed. B2-expressing virus showed enhanced killing in the majority of cancer cell lines tested, including M14 and 786-O cells (Fig. 2a). In our screen, we also included an additional virus variant encoding vaccinia Copenhagen virus VP55, a different VSR [26], which similarly enhanced virus-mediated cell killing in our studies (Fig. 2a).

To determine whether VSV∆51-B2 could affect virus production, we assessed viral replication at multiple time points in M14 and 786-O cells, and found that VSV∆51-B2 significantly enhanced replication over time, relative to VSV∆51-GFP (Fig. 2b). Further, we infected GM38 fibroblasts with VSVΔ51-B2 or VSVΔ51-GFP to investigate if B2 expression affects virus replication in healthy cells, and found that VSVΔ51-B2 infection did not significantly increase viral cytotoxicity at an MOI of 1 (Additional file 1: Figure S2A).

### VSV∆51-B2 prevents VSV genome cleavage in cancer cells

To determine whether B2 protects VSV from genome cleavage, we performed small-RNA deep-sequencing on VSVΔ51-B2 infected cancer cells similar to the experiment in Fig. 1. We showed that B2 expression in VSVΔ51 virus abrogates genomic cleavage as 22-mer vsRNAs are no longer prominent in different cell lines (Fig. 2c and Additional file 1: Figure. S2B). Interestingly, VSVΔ51-B2 derived vsRNAs display a bias towards positive strand reads compared to VSVΔ51 vsRNAs in cancer cells (Fig. 2c and Additional file 1: Figure S2B). Since the viral positive strand consists of viral mRNAs and positive sense genome copies, a larger bias for positive sense vRNAs suggests more efficient mRNA transcription in our VSVΔ51-B2 virus. In addition, we infected M14 and 786-O cells with VSVΔ51-GFP or VSVΔ51-B2 and showed that the expression level of virus genomes for each sample was enhanced in response to VSVΔ51-B2, relative to VSVΔ51-GFP (Fig. 2d). Taken together, these data suggest that VSV∆51-B2 inhibits direct cleavage of the viral genome and host RNA processing pathways.

### VSV∆51-B2 and the type I IFN response

**Fig. 3:**
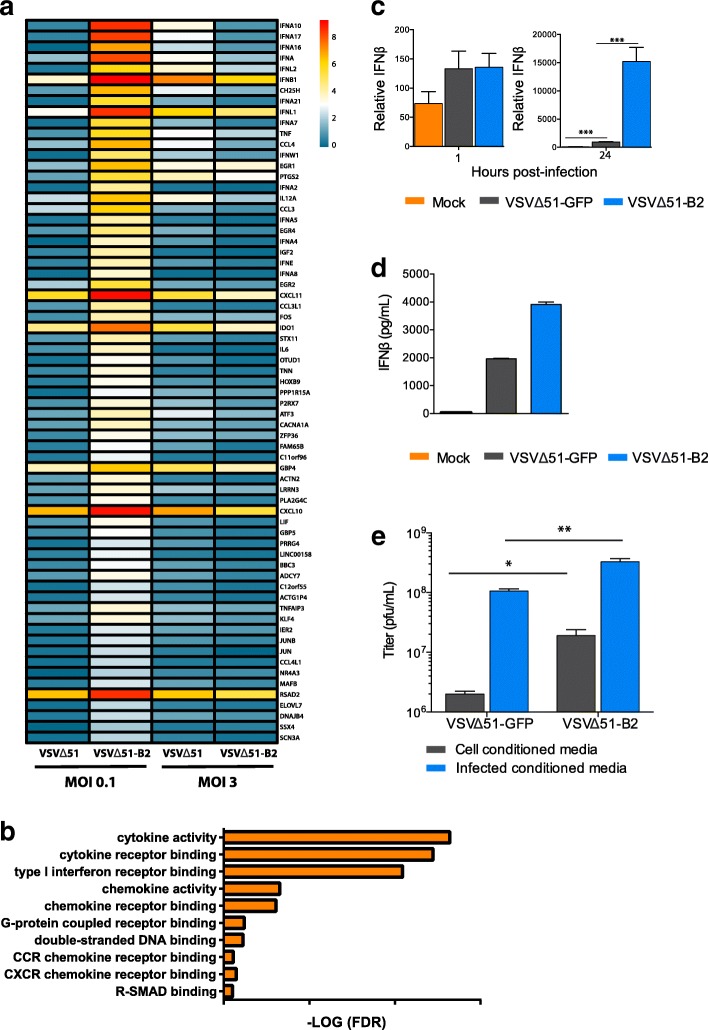
VSV∆51-B2 modulates IFN response and cytokine production. **a** Microarray analysis of M14 cells infected with VSVΔ51-GFP or VSVΔ51-B2 at low and high MOI as indicated. **b** Enrichment of cytokine and cytokine activity in the microarray, associated with an IFN response. **c** qPCR analysis of IFN-β expression of 786-O cells infected for various times. IFN-β levels were normalized to GAPDH levels within each sample. **d** ELISA for IFN-β from supernatants of 786-O cells infected with VSVΔ51-GFP or VSVΔ51-B2 at an MOI of 0.1 for 24 h. **e** Virus outputs of VSVΔ51-GFP and VSVΔ51-B2 obtained from 786-O cells pre-treated with vaccinia Copenhagen virus conditioned-media. Virus-cleared supernatants from HeLa cells that were infected with vaccinia Copenhagen virus at an MOI of 1 for 48 h or left uninfected were transferred onto 786-O cells prior to infection with VSVΔ51-GFP or VSVΔ51-B2 for 48 h. NS: *P* > 0.1, *P < 0.1, **P < 0.01, ***P < 0.001, using Student’s *t*-test. Only significantly different pairs are indicated on the figure

To characterize the effect of B2 expression on mammalian cancer cells at a transcriptome level, we performed a microarray analysis on samples from M14 cells infected with either VSV∆51-GFP or VSV∆51-B2. Our results show that at a low MOI, VSV∆51-B2 virus induced the expression of a variety of immune related genes, which were not affected by VSV∆51 infection (Fig. 3a). Through GO-term analysis, we detected an upregulation of genes by at least four-fold in response to VSV∆51-B2, but not VSV∆51-GFP. We also showed enrichment of cytokine and cytokine activity predominantly associated with an IFN response (Fig. 3b). Interestingly, at high MOI, most immune genes upregulated by VSV∆51-B2 at low MOI remain unchanged with few visible differences between the viruses (Fig. 3a).

Given that the IFN response is an important antiviral mechanism in mammalian cells, we investigated the potential impact of B2 on IFN responsiveness by qPCR after infection of 786-O cells. Consistent with our microarray results, we observed a significant increase in IFN-ß levels 24 h post-infection with VSV∆51-B2 relative to the control virus (Fig. 3c). We also showed that VSV∆51-B2 enhances IFN-ß secretion in 786-O cells, relative to VSV∆51-GFP, by ELISA (Fig. 3d). Lastly, we investigated if the production of VSV∆51-B2 could be further enhanced by blocking the IFN pathway. We have previously shown that B19R, a soluble type I IFN scavenger expressed by vaccinia Copenhagen virus, enhances VSV∆51 production [26]. In order to block the antiviral effect of the IFN that would be produced in response to VSV∆51 infection, we generated conditioned-media from vaccinia virus-infected HeLa cells and pre-treated 786-O cells with B19R-containing media. We found that viral titers were significantly higher for VSV∆51-B2 with both control media and vaccinia Copenhagen virus conditioned-media compared to VSV∆51-GFP (Fig. 3e); however, the absolute increase in virus titers after exposing the cells to vaccinia Copenhagen virus-conditioned media was similar for both VSVΔ51-GFP and VSVΔ51-B2. Although induction of antiviral RNAi has been shown in mature mammalian cells [27, 28], our data suggest that the RNAi-based pathway is an IFN-independent antiviral mechanism in cancer cells as B2 expression alone enhances viral titers, which can be further enhanced by blocking the IFN response.

### Virus-mediated B2 expression enhances replication and cytokine production in vivo

**Fig. 4:**
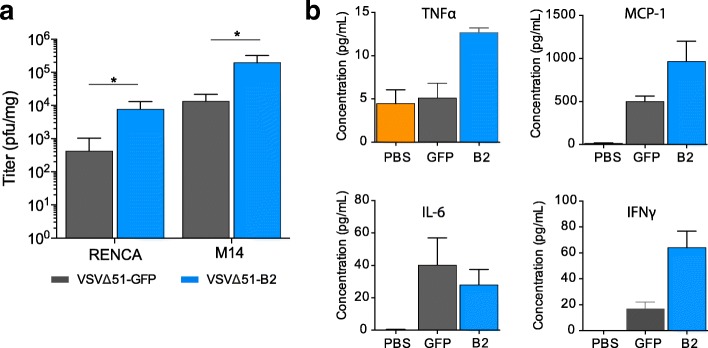
VSV∆51-B2 enhances replication and cytokine levels within in vivo tumour models. **a** Viral titers obtained 24 hpi from subcutaneous M14 or RENCA tumours. Virus was administered intratumourally at a dose of 1E9 pfu of VSVΔ51-GFP or VSVΔ51-B2. NS: *P* > 0.1, *P < 0.1, **P < 0.01, ***P < 0.001, using Student’s *t*-test. Only significantly different pairs are indicated on the fig. **b** Serum levels of TNF-α, MCP-1, IL-6 and IFN-γ from RENCA tumour-bearing C57BL/6 mice. Virus was administered intratumourally at a dose of 1E9 pfu of VSVΔ51-GFP or VSVΔ51-B2 and serum collected 24 hpi. NS: *P* > 0.1, *P < 0.1, **P < 0.01, ***P < 0.001, using Student’s *t*-test. Only significantly different pairs are indicated on the figure

To establish an in vivo mouse model, we first screened RENCA mouse renal carcinoma cells in vitro to determine whether VSV∆51-B2 enhanced cytotoxicity. We showed that, as observed using human cell lines, RENCA cells were more efficiently killed by the B2-expressing virus (Additional file 1: Figure S2C). In vivo, we subsequently tested the RENCA cell line which is syngeneic to Balb/c mice. In addition, we used the human M14 melanoma cell line as a xenograft model in nude mice. For both models, VSV∆51-B2 titers from subcutaneous tumours harvested 24 h post-intratumoural viral injection were significantly higher compared to VSV∆51-GFP (Fig. 4a). Consistent with our microarray analysis, the IFN-γ, TNF-α and MCP-1 concentrations from the serum of the RENCA tumour-bearing Balb/c mice were significantly increased for the VSV∆51-B2 treated mice compared to VSV∆51-GFP treated mice (Fig. 4b). In contrast, Il-6 concentrations did not significantly increase (Fig. 4b). Biodistribution analyses following intravenous administration revealed unchanged amounts of virus obtained from the different organs for VSV∆51 and VSV∆51-B2 (Additional file 1: Figure S2D-E).

## Discussion

In this study, we demonstrate that B2 expression is sufficient to enhance VSV∆51 replication and cytotoxicity (Fig. 1-2) in mammalian cancer cells. Our screen of human cancer cells demonstrated enhanced efficacy of VSV∆51-B2 in killing the majority of cancer cell lines tested. The enhanced cytotoxic ability of VSV∆51-B2 suggests that RNAi may be an important factor hindering virus replication in resistant cancer cells. It is important to note that the cell lines in which there was no difference in cytotoxicity were the most sensitive to viral infection (Fig. 2), suggesting that the lack of improvement could potentially be attributed to the already maximal virus production by these cells. An antiviral RNAi system may still function in these cells but may only be apparent in certain conditions, such as within the tumour microenvironment where many factors come together and create additional barriers to infection.

Interestingly, virus variants encoding B2 and VP55, two VSRs that impair the RNAi response by different mechanisms, show the same improvement in killing ability for all cell lines tested (Fig. 2). The mechanism of B2 involves binding of small RNA fragments which could either prevent their processing by Dicer or loading into RISC. On the other hand, VP55 polyadenylates miRNAs which targets them for degradation [29]. Given that both VSRs improve VSV∆51-mediated killing to the same extent, this suggests that inhibition of the RNAi pathway improves virus replication regardless of the mechanism through which inhibition is achieved. VP55 does not polyadenylate all small RNAs and key features such as the presence of a 2’O methyl group protect a subset of small RNAs from degradation [29]. Notably, vsRNAs have been previously shown to be 2’O methylated, which protects them from degradation [30]. This may be advantageous for cell lines in which direct viral genome cleavage occurs as ideally, cleaved genome fragments can provide additional protection through targeting homologous viral genomes and transcripts.

To begin exploring the mechanism of B2 on VSV∆51 replication, we investigated the potential impact of B2 on the IFN response. A number of cellular proteins such as Toll-like receptor 3, retinoic acid inducible gene I, 2′-5′ oligoadenylate synthetase and protein kinase R recognize dsRNA and trigger a potent antiviral immune response [31]. Therefore, B2 may sequester the dsRNA substrates of these antiviral factors or interact with these proteins to prevent the sensing of dsRNA. As such, we investigated the effects of B2 on the IFN response, which is downstream of these pathways. We demonstrate that B2 does not suppress but actually significantly increases IFN-ß production (Fig. 3) compared to control virus, which is likely a result of enhanced replication. More specifically, at low MOI we detect an upregulation of immune genes with VSV∆51-B2 due to the ability of the VSV∆51-B2 virus to replicate in M14 cells and establish a successful infection which triggers a more robust IFN response (Fig. 3). However, at high MOI we do not see upregulation of immune genes by either VSV∆51-B2 or VSV∆51-GFP virus (Fig. 3). Higher MOI is often used to overcome resistance to infectivity and often leads to faster cell death, suggesting there was insufficient time to mount a type I IFN response as most, if not all, cells were infected in the first round of replication. Of note, low MOI levels are more comparable to in vivo systems, in which VSV∆51-B2 virus may be more immunogenic. This prediction is in line with higher cytokine (IFN-γ, TNF-α and MCP-1) levels found in the serum of tumour bearing VSV∆51-B2 treated immune-competent mice (Fig. 4).

Additionally, our vaccinia Copenhagen virus-conditioned media transfer experiments demonstrated that the blocking of type I IFN did not further enhance VSV∆51-B2 replication compared to VSV∆51-GFP. This is an indirect way to neutralize IFN-1 [26] and further suggested that IFN and B2 have different mechanisms of action (Fig. 3). Consistent with this notion, a similar induction of IFN-stimulated genes was observed in wildtype and RNAi-defective mouse embryonic fibroblasts [27]. In addition, suppression of RNAi by Nodamura virus protein B2 protein does not change the expression levels of IFN-stimulated genes in infected mice [17]. Importantly, our data do not eliminate the possibility of IFN-stimulated miRNAs limiting VSV∆51 efficacy. Despite stimulating a type I IFN response, VSV∆51-B2 has enhanced replication, suggesting that B2 expression is sufficient to overcome the antiviral response it stimulates.

One study has shown production of abundant influenza viral siRNAs in IFN-competent A549 cancer cells [27], but the existence of antiviral RNAi in cancer cells remains largely unexplored. We were able to detect viral genome fragments (vsRNAs) following infection with VSV∆51 in a number of cell lines (Fig. 1 and Additional file 1: Figure S1), suggesting that viral genome cleavage is occurring. This may be facilitated by RNAi machinery being repurposed during viral infection. Interestingly, VSV∆51-B2 infection resulted in a decrease in the percentage of vsRNAs (Fig. 2 and Additional file 1: Figure S2), suggesting the efficient prevention of this antiviral mechanism. A number of recent reports support the notion of antiviral genome cleavage in mammalian cells. It has been demonstrated that infection with RNA viruses can trigger the production of viral siRNAs, presumably as a result of direct virus genome cleavage [17, 32]. The fact that many mammalian viruses encode VSRs, further supports the concept of a mammalian RNAi system. For example, Influenza A encodes the NS1 protein [33, 34], Ebola encodes VP35 [35, 36], HIV-1 encodes Tat [37, 38] and vaccinia Copenhagen virus encodes VP55 [29]. These proteins all have VSR-like functions, which suggests an evolutionary advantage of blocking antiviral RNAi.

It is possible that both direct inhibition of viral genome cleavage and inhibition of cellular antiviral miRNA production are co-occurring within certain cancer cell lines, as we observe changes in both VSV-specific and total RNA read length distributions (Additional file 1: Figure S1 and S2 and data not shown). The potential interplay between viruses and infected host cell miRNAs is a concept that is supported by several studies. For example, miR-29 has been reported to bind the 3’ UTR of HIV mRNA which inhibits its translation and results in sequestering the mRNA into processing bodies [39]. IFN-ß itself induces transcription of a number of miRNAs in hepatocytes that are complimentary to the hepatitis C virus genomic RNA and inhibit viral replication [40]. In fact, activation of the IFN pathway has been shown to lead to the upregulation of a number of miRNAs including miR-1 [40–42], miR-129 [43], miR-146 [42] and miR-155 [42, 44, 45], some of which likely function to control infection. Perhaps most relevant to our study, 2 miRNAs (miR-24 and miR-93) have been previously shown to directly target the VSV genome and limit VSV replication [46]. Our results show that VSV∆51-B2 infection leads to the downregulation of both of these miRNAs, providing a potential explanation for the increased virus production using the B2 virus.

Overall, we demonstrate a novel role of the RNAi pathway as an intrinsic antiviral mechanism in cancer cells and how RNAi inhibition may be used to improve OV replication. Mechanistically, inhibition of direct viral genome cleavage and/or modulation of miRNA processing contribute to enhancing VSV∆51 infection in a cell line specific manner. This work provides insight into the basic biology of viral defense mechanisms in cancer and promises to improve current OV therapies by tailoring viruses to overcome alternative antiviral mechanisms.

## Methods

### Cell lines and culture

All cell lines were purchased from the American Type Culture Collections (Manassas, VA). Mammalian cells were cultured in Dulbecco’s modified Eagle’s medium (DMEM) (Corning cellgro, Manassas, VA) or RPMI-1640 (K562, OVCAR3, OVCAR4, OVCAR8) (Corning cellgro, Manassas, VA) supplemented with 10% fetal bovine serum (FBS) (Sigma life science, St-Louis, MO) and maintained at 37°C with 5% CO_2_. *Drosophila melanogaster* Schneider line 2 (S2) cells were cultured in SF900II serum-free medium (Invitrogen) at 25 °C under atmospheric pressures.

### DNA constructs and viral constructs

The pcDNA3.1-puro B2 (Nodamura gene) plasmid used for generating the B2-expressing stable cell lines was made available by the Christopher Sullivan lab (Addgene plasmid # 17228). The pEGFP-N1 plasmid (Catalog # 6085–1) was purchased from Clontech (Moutain View, CA).

The B2 gene was amplified by PCR of the Nodamura virus genome. The primers were designed to include the XhoI and NheI restriction sites as well as to insert a 6× histidine tag at the 5′ end of the B2 sequence. The digested PCR fragment was cloned into the XhoI and NheI digested VSVΔ51 backbone, as previously described. The primer pairs for inserting B2 into the VSV backbone are listed in Additional file 2: Table S1.

VP55 was PCR amplified from the Copenhagen strain of vaccinia virus and subcloned into pcDNA3.1 with an N-terminal Flag epitope. Flag-VP55 was subsequently PCR amplified and cloned into VSVΔ51M using the same strategy.

### Transfection and selection of cell lines

M14 and 786-O cells were transfected using Lipofectamine 2000 (Invitrogen, Carlsbad, CA) according to manufacturer instructions. Briefly, cells were plated in 6 well format 1 day prior to transfection. Plasmid and Lipofectamine reagent were incubated for 20 min and then added to plated cells in OptiMEM (Thermoscientific, Waltham, MA). 24 h post-transfection, medium was replaced by DMEM with 10% FBS and cultured for 48 h. Cells were then subjected to drug selection by the addition of Geneticin (800 μg/mL) (Thermo Fisher, Carlsbad, CA). Cells were expanded and GFP- or YFP-positive cells were sorted twice by FACS (MoFlo Astrios).

### Virus quantification

Viral titers were obtained by plaque assays. Serial dilutions of the samples were prepared in serum-free DMEM. The dilutions were then transferred to monolayers of Vero cells and incubated at 37 °C for 1 h. After the incubation, cells were overlaid with 0.5% agarose in DMEM supplemented with 10% FBS. Plates were incubated for 24 h at 37 °C with 5% CO_2_ and plaques were counted.

### Virus rescue and purification

Virus rescues were performed as previously described. Vero cells were infected with T7 polymerase-expressing vaccinia Copenhagen virus at an MOI of 3. Following a 2 h incubation, media was removed and cells were transfected with T7-driven plasmids encoding VSV N, P, and L genes as well as the VSV∆51-B2 backbone. Supernatants collected 48 h post-transfection were passed through a 0.22 μm filter (MillexGP, Carrigtwohill, Ireland) to remove vaccinia Copenhagen virus.

For expansion and purification of the viral preparations, Vero cells were infected at an MOI of 0.001 and culture supernatants were collected 24 h post-infection. Supernatants were then filtered through a 0.2 μm bottle top filter (Millipore, Etobicoke, Canada) and centrifuged at 30,100 *g* for 90 min. The supernatant was discarded and pelleted virus was resuspended in Dulbecco’s phosphate-buffered saline (Corning cellgro, Manassas, VA). Purified virus was kept at − 80°C.

### Deep sequencing of vsRNAs

Total RNA was extracted with TRIzol reagent (Invitrogen) according to manufacturer instructions. Library preparation for Illumina sequencing was performed (TCAG DNA Sequencing Facility, Toronto, ON). Briefly, RNA was enriched for 15–25 nt sizes before strand-specific, small-RNA library preparation and 50 bp single end read sequencing. Adapter trimming was done with Trimmomatic [47] following default parameters. Before read mapping, VSV∆51 genome was constructed from the VSV reference genome (NC_001560), manually edited to delete the 51st methionine amino acid in the M gene. Reads were mapped to VSV∆51 genome using bbmap.sh script from the BBMap toolkit (http://sourceforge.net/projects/bbmap) with a minimum alignment identity of 100%. SAMtools was used to separate positive sense mapping from bbmap produced sam files [48]. Lastly, positive sense reads were analyzed for size distribution using the reformat.sh script from the BBMap toolkit.

### Western blotting

Cell pellets were lysed on ice for 30 min using complete protease inhibitor cocktail (Roche, Mississauga, Ontario, Canada) supplemented lysis buffer (1% NP40, 150 mM NaCl, 5 mM EDTA, 50 mM Tris pH 7.4). Lysates were centrifuged for 10 min at 16,000 *g* and cleared supernatants were mixed with dithiothreitol-supplemented loading buffer (250 mM Tris-HCl pH 6.8, 10% SDS, 30% glycerol, 5% β-Mercaptoethanol, 0.02% bromophenol blue). The samples were migrated on Bio-Rad Mini Protean 4–15% TGX Protein Gels (Bio-Rad, Mississauga, ON) and transferred onto PVDF membranes (GE Healthcare, Buckinghanshire, UK) prior to blocking with 5% skim milk powder (Oxoid Ltd., Basingstoke, UK) in Tris-buffered saline (TBS)with 0.1% Tween-20. The membranes were proofed using specific rabbit antibodies for 6× His tag (Abcam, Cambridge, UK), VSV (polyclonal anti-VSV serum for hyperimmune rabbits) [49]. Rabbit anti-GAPDH (Abcam, Cambridge, UK) and rat anti-tubulin (Novus Biologicals, Oakville, ON) antibodies were used as loading controls. Membranes were then probed with a horse radish peroxidase-coupled goat anti-rabbit secondary antibody (Millipore, Etobicoke, Canada) or goat anti-rat secondary antibody (Life Technologies, Carlsbad, CA) and the signal was revealed using Amersham ECL Western Blotting Detection Reagent (GE Healthcare, Buckinghamshire, UK). The gels were analyzed using FluorChem FC2 (Alpha Innotech, San Leandro, CA).

### Cell viability assay

Relative metabolic activity of cells was used as a readout of cell viability and was determined using alamarBlue reagent (Bio-Rad, Mississauga, Ontario, Canada). The assays were performed according to manufacturer instructions. Briefly, cells were plated in 96-well plates and infected with the different viruses 24 h later. 48 h after virus infection, alamarBlue was added to each well to a final concentration of 1:10. The samples were incubated 1 to 5 h and the fluorescence readings (excitation and emission wavelengths of 530 nm and 590 nm, respectively) were taken using a Fluoroskan Ascent FL (Thermo Labsystems, Beverly, MA).

### Quantitative PCR

For miRNA qPCRs, RNA was extracted from infected cell pellets using TRIzol reagent (Life Technologies, Carlsbad, CA) according to the manufacturer instructions. The RNA concentration and purity was assessed using a NanoDrop ND-1000 spectrophotometer (Thermoscientific, Waltham, MA) prior to reverse transcription using Quanta miRNA cDNA synthesis kit (Gaithersburg, MD).

For all other qPCRs, RNA was extracted using RNAeasy RNA extraction kit (QIAGEN, Toronto, ON, Canada) according to the manufacturer’s instructions. The RNA concentration and purity was assessed using a NanoDrop ND-1000 spectrophotometer (Thermoscientific, Waltham, MA) prior to reverse transcription using RevertAid H Minus First Strand cDNA Synthesis kit (Thermoscientific, Waltham, MA).

qRT -PCR was performed on the non-pooled triplicate samples. Following conversion to cDNA by Superscript RT II (Invitrogen, Carlsbad, CA), qRT-PCR was carried out using Sybergreen (Invitrogen) according to the manufacturer’s instructions. Analyses were performed on a Rotor-Gene RG-3000A machine (Corbett Research, Mortlake, AU) according to the manufacture instructions. The primer pairs specific for various gene products used in our experiments are listed in Additional file 2: Table S1. qRT-PCR measurements were normalized to the *U6* or *GAPDH* house-keeping genes for miRNA or RNA transcripts, respectively, using the Pfaffl method [50].

### Microarray

Monolayers of M14 cells were treated at an MOI of 0.1 or 3 for 24 h with either VSV∆51 or VSV∆51 encoding B2 gene virus Total RNA was extracted with TRIzol reagent (Invitrogen) according to the manufacturer instructions. Experimental triplicate total RNA samples were processed by The Center for Applied Genomics at The Hospital for Sick Children for microarray analysis on a Human Prime View chip. Raw files were analyzed using the Transcriptome Analysis Console v3.0 (Affymetrix) software. Normalized transcript expression values further processed with R. Heatmaps were produced using the R package “pheatmap” v1.0.8. GO Term Enrichment analysis (http://CRAN.R-project.org/package=pheatmap) was performed using the online EnrichR tool [51]. Genes selected for enrichment analysis are the subset of genes upregulated by the expression of the B2 gene in VSV∆51 by at least 4-fold.

### ELISA

The concentration of IFN-β was determined using the human IFN- β ELISA kit (R&D Systems, Minneapolis, MN) according to the manufacturer’s instructions.

### Supernatant transfer experiments

Vaccinia stocks were propagated in U-2 OS cells and cell-associated virus was collected by repeat [3] freeze–thaw cycles. Purification of viral stocks was done by centrifugation at 20,700 *g* through a 36% sucrose cushion (in 1 mM Tris) before resuspension in 1 mM Tris, pH 9.

To generate infected cell conditioned media, U-2 OS cells were either mock treated or infected with VVdd-mCherry at a multiplicity of 10 PFU/cell for 24 h, harvested and then pelleted by centrifugation. Supernatants were collected and passed through a 0.22 μm filter to eliminate cell-free vaccinia virions. To test for factors enhancing VSV infectivity, tumour cell monolayers were pre-treated for 2 h with conditioned U-2 OS supernatant. Tumour cells were then infected with VSV in the presence of conditioned medium.

### In vivo experiments and tumour models

6–8 weeks old female Balb/c or nude mice (Charles River Laboratories, Wilmington, MA) were used. For the Balb/c mice 5 × 10^5^ RENCA tumour cells were implanted subcutaneously 21 days prior to treatment. For the nude mice 1 × 10^8^ M14 tumour cells were implanted subcutaneously 14 days prior to treatment. A single intratumoural injection of 1E8 PFU of VSVΔ51-GFP or VSVΔ51-B2 was performed. Tumours were harvested 24 h post-treatment, weighed and homogenized in PBS using a Powergen 125 tissue homogenizer (Fischer Scientific, Hampton, New Hampshire). Serial dilutions of tumour homogenates were tittered to obtain intratumoural titer data. For biodistribution experiments, naïve Balb/c mice were treated with a single intravenous injection of 1E8 PFU of VSVΔ51-GFP or VSVΔ51-B2. Mice were sacrificed 24 h or 48 h post-treatment and organs were harvested, frozen and homogenized in PBS as above. All experiments were approved by the University of Ottawa animal care and veterinary services (ME-2258).

Serum was obtained upon centrifugation of blood samples collected using lithium-heparin coated capillary tubes (Sarstedt, Newton) at 16000 *g* for 5 min. The concentrations of IFNγ, TNFα, MCP-1 and IL-6 were measured using a mouse inflammation cytometric bead array kit (BD Biosciences, San Jose, CA) according to manufacturer instructions. The results were acquired on a LSR Fortessa flow cytometer and analyzed using the FCAP array software (BD Biosciences).

## Additional files

Additional file 1: **Figure S1.** B2 selectively enhances VSV∆51 replication in other cancer cells. (A) MCF7, HT-29, or SF-295 cells were infected with VSVΔ51 virus and small-RNA deep sequencing was performed. Virus-derived small RNAs have a length bias towards 22-mers. The enrichment for 22-mers is indicated for positive strand vsRNAs. (B) Fluorescence microscopy images of M14 or 786-O cells stably expressing EGFP-B2 or fluorescently-tagged empty vector (mock control). **Figure S2.** VSVΔ51-B2 does not enhance viral replication in non-cancer healthy cells or alter biodistribution in various organs. (A) Relative metabolic activity of GM38 fibroblasts infected with VSVΔ51-GFP or VSVΔ51-B2 for 48 h at an MOI of 1. The results are expressed as a percentage of the signal obtained compared to mock treatment. NS: *P* > 0.1, *P < 0.1, **P < 0.01, ***P < 0.001, using Student’s *t*-test. Only significantly different pairs are indicated on the fig. (B) We performed small-RNA deep-sequencing using MCF7, HT-29, or SF-295 cells infected with VSVΔ51-B2 at an MOI of 0.1 for 18 h. B2 expression in VSVΔ51 virus abrogates genomic cleavage as 22-mer vsRNAs are no longer prominent. VSVΔ51-B2 derived vsRNAs display a bias towards positive strand reads in M14 and 786-O cells. (C) Relative metabolic activity of RENCA cells infected with VSVΔ51-GFP or VSVΔ51-B2 for 48 h at an MOI of 1. The results are expressed as a percentage of the signal obtained compared to mock treatment. (D&E) Biodistribution of VSVΔ51-B2 in tumour-bearing C57BL/6 mice. Viral titers obtained from organs of tumour-bearing C57BL/6 mice, D] 24 or E] 48 hpi. Virus was administered intravenously at a dose of 1E9 pfu of VSVΔ51-GFP or VSVΔ51-B2. For organs where virus was undetectable, the titer was considered to be the value of the limit of detection of titering for this assay (5E1 pfu/organ). NS: *P* > 0.1, *P < 0.1, **P < 0.01, ***P < 0.001, using Student’s *t*-test. (PDF 5336 kb)

Additional file 2: **Table S1.** List of primers used in qRT-PCR assays. (DOCX 14 kb)

## Abbreviations

IFN: Interferon
miRNA: MicroRNA
OV: Oncolytic virus
qPCR: Quantitative PCR
RISC: RNA-induced silencing complex
RNAi: RNA interference
VSR: Viral suppressors of RNAi
vsRNA: Virus-derived small RNAs
VSV: Vesicular stomatitis virus
