# Supplementary figures and images for: Enhanced susceptibility of cancer cells to oncolytic rhabdo-virotherapy by expression of Nodamura virus protein B2 as a suppressor of RNA interference
Source: J Immunother Cancer. 2018 Jun 19;6:62. doi: 10.1186/s40425-018-0366-2 (PMC6008949; doi:10.1186/s40425-018-0366-2)

**a**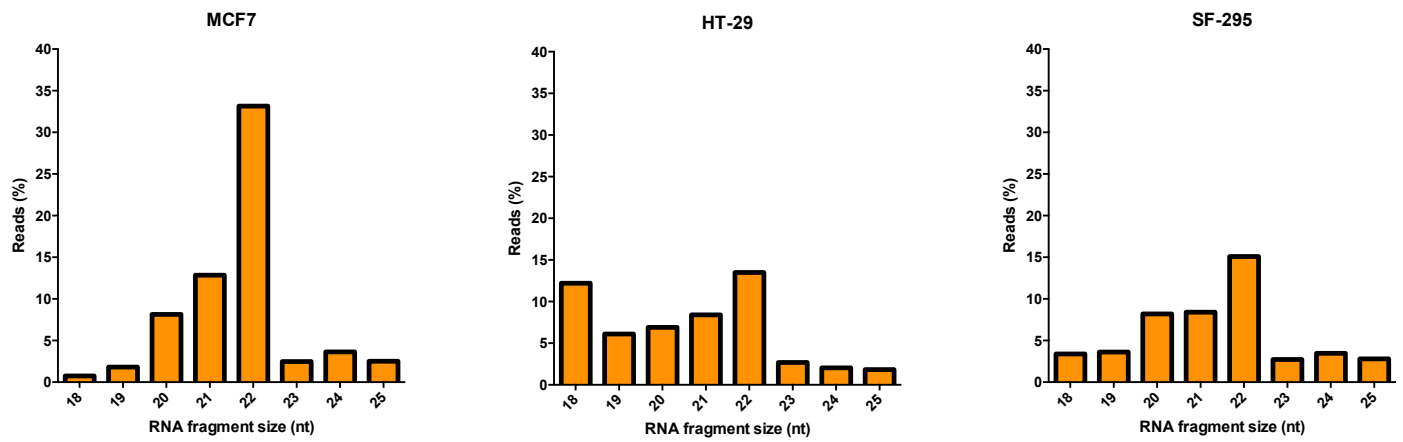**b**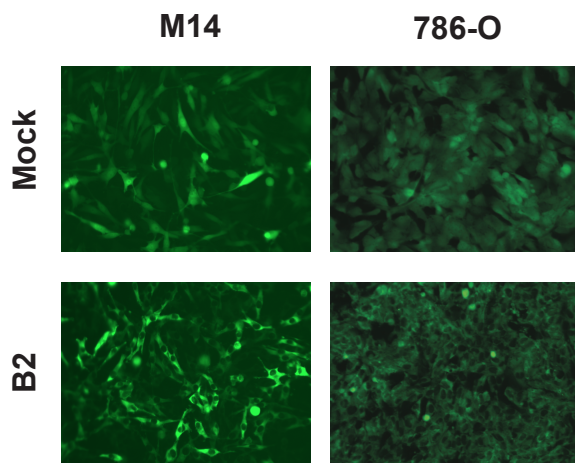

**a**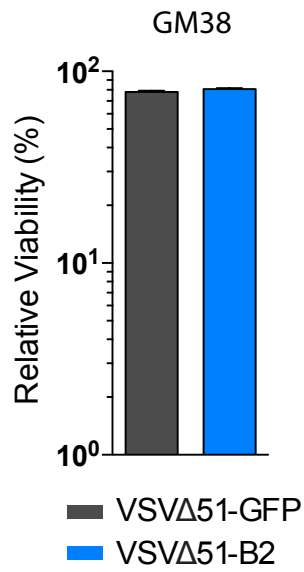**b**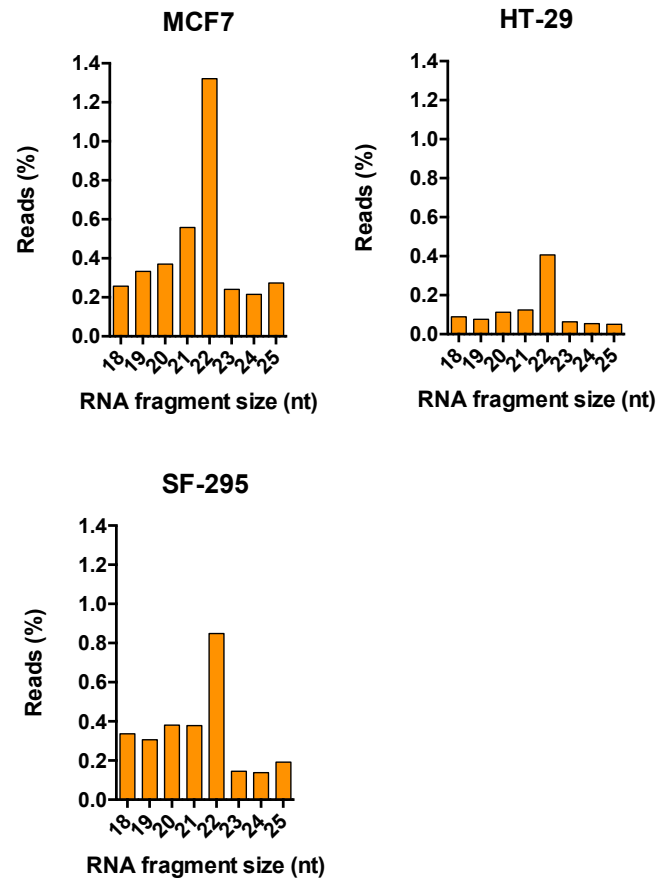**c**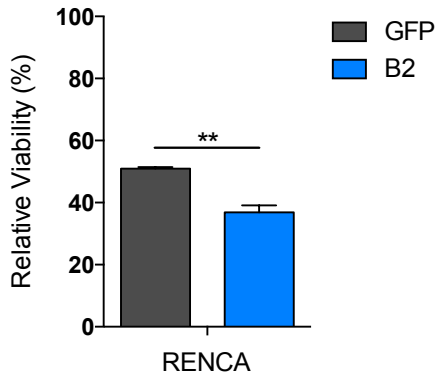**d**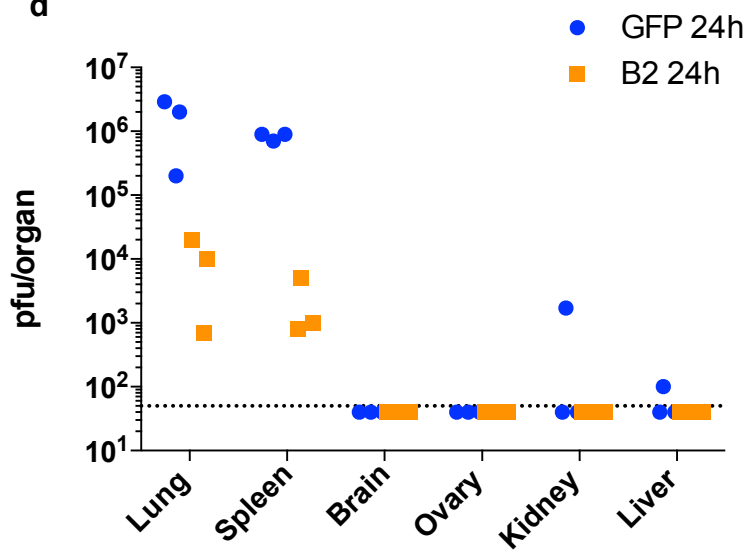**e**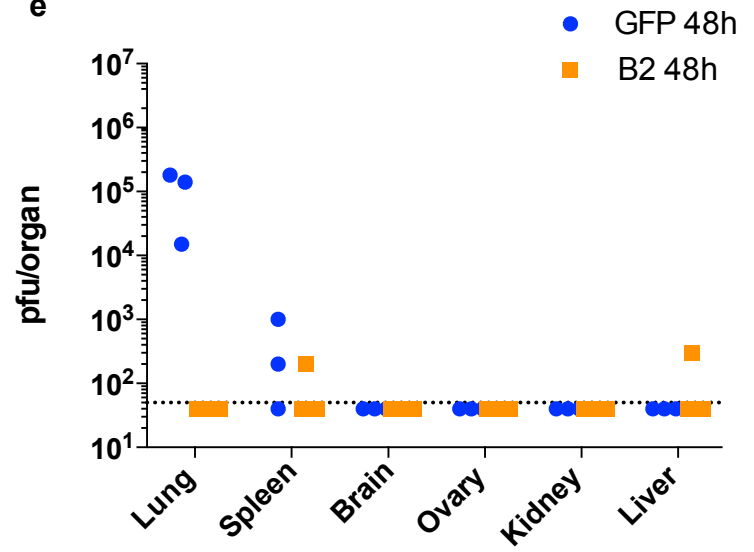

Supplement: Supplementary file 1 — Figure S1. B2 selectively enhances VSV∆51 replication in other cancer cells. (A) MCF7, HT-29, or SF-295 cells were infected with VSVΔ51 virus and small-RNA deep sequencing was performed. Virus-derived small RNAs have a length bias towards 22-mers. The enrichment for 22-mers is indicated for positive strand vsRNAs. (B) Fluorescence microscopy images of M14 or 786-O cells stably expressing EGFP-B2 or fluorescently-tagged empty vector (mock control). Figure S2. VSVΔ51-B2 does not enhance viral replication in non-cancer healthy cells or alter biodistribution in various organs. (A) Relative metabolic activity of GM38 fibroblasts infected with VSVΔ51-GFP or VSVΔ51-B2 for 48 h at an MOI of 1. The results are expressed as a percentage of the signal obtained compared to mock treatment. NS: P > 0.1, *P < 0.1, **P < 0.01, ***P < 0.001, using Student’s t-test. Only significantly different pairs are indicated on the fig. (B) We performed small-RNA deep-sequencing using MCF7, HT-29, or SF-295 cells infected with VSVΔ51-B2 at an MOI of 0.1 for 18 h. B2 expression in VSVΔ51 virus abrogates genomic cleavage as 22-mer vsRNAs are no longer prominent. VSVΔ51-B2 derived vsRNAs display a bias towards positive strand reads in M14 and 786-O cells. (C) Relative metabolic activity of RENCA cells infected with VSVΔ51-GFP or VSVΔ51-B2 for 48 h at an MOI of 1. The results are expressed as a percentage of the signal obtained compared to mock treatment. (D&E) Biodistribution of VSVΔ51-B2 in tumour-bearing C57BL/6 mice. Viral titers obtained from organs of tumour-bearing C57BL/6 mice, D] 24 or E] 48 hpi. Virus was administered intravenously at a dose of 1E9 pfu of VSVΔ51-GFP or VSVΔ51-B2. For organs where virus was undetectable, the titer was considered to be the value of the limit of detection of titering for this assay (5E1 pfu/organ). NS: P > 0.1, *P < 0.1, **P < 0.01, ***P < 0.001, using Student’s t-test. (PDF 5336 kb) [file 40425_2018_366_MOESM1_ESM.pdf]
