# Supplementary material for: Enhanced susceptibility of cancer cells to oncolytic rhabdo-virotherapy by expression of Nodamura virus protein B2 as a suppressor of RNA interference
Source: J Immunother Cancer. 2018 Jun 19;6:62. doi: 10.1186/s40425-018-0366-2 (PMC6008949; doi:10.1186/s40425-018-0366-2)
Supplement: Supplementary file 2 — Table S1. List of primers used in qRT-PCR assays. (DOCX 14 kb) [file 40425_2018_366_MOESM2_ESM.docx]

**Table S1.** List of primers used in qRT-PCR assays.

*qPCR primers for miRNA*

has-mir24-1-5p TGCCTACTGAGCTGATATCAGT

hsa-mir93-5p CAAAGTGCTGTTCGTGCAGGTAG

hsa-mir378-3p ACTGGACTTGGAGTCAGAAGGC

hsa-mir125a-5p TCCCTGAGACCCTTTAACCTGTGA

hsa-mir142-5p CATAAAGTAGAAAGCACTACT

hsa-mir155-5p TTAATGCTAATCGTGATAGGGGT

hsa-let7a-5p TGAGGTAGTAGGTTGTATAGTT

hsa-mir16-5p TAGCAGCACGTAAATATTGGCG

hsa-mir31-5p AGGCAAGATGCTGGCATAGCT

hsa-mir1-3p TGGAATGTAAAGAAGTATGTAT

hsa-mir196b-5p TAGGTAGTTTCCTGTTGTTGGG

hsa-mir146a-5p TGGAATGTAAAGAAGTATGTAT

hsa-mir423-5p TGAGGGGCAGAGAGCGAGACTTT

hsa-mir128-3p TCACAGTGAACCGGTCTCTTT

hsa-U6 GTGCTCGCTTCGGCAGCACATATA

*qPCR primers for human IFN pathway*

IFN-β-F CTCTCCTGTTGTGCTTCTCC

IFN-β-R GTCAAAGTTCATCCTGTCCTTG

*qPCR primers for human GAPDH*

GAPDH-F ACACATTGGGGGTAGGAACA

GAPDH-R AACTTTGGCATTGTGGAAGG

*PCR primers for inserting B2 into the VSV backbone*

B2-F: TATTCTCGACATGCATCATCACCACCACCATACAAACATGTCATGCGCTTAC

B2-R: ATTTGCTAGCATCACTCATTTACCACGCCC

*PCR primers for VSV*

VSV-F: GGA ATC TGG CTG CAG CAA AG

VSV-R: ATG CAA AGA TGG ATA CCA AC
